# Is the Sentinel Lymph Node Biopsy Safe and Accurate After Previous Surgery for Vulvar Squamous Cell Carcinoma? A Systematic Review

**DOI:** 10.3390/cancers17040673

**Published:** 2025-02-17

**Authors:** Luigi Della Corte, Dominga Boccia, Federica Cinque, Cristina Pisano, Giuseppe Gullo, Valentina Billone, Stefano Restaino, Giuseppe Vizzielli, Pierluigi Giampaolino, Giuseppe Bifulco

**Affiliations:** 1 Department of Neuroscience, Reproductive Sciences and Dentistry, School of Medicine, University of Naples “Federico II”, 80131 Naples, Italy; 2 Department of Public Health, School of Medicine, University of Naples “Federico II”, 80131 Naples, Italy; cinquefede5@gmail.com (F.C.); cristinapisano@outlook.com (C.P.); pgiampaolino@gmail.com (P.G.); giuseppe.bifulco@unina.it (G.B.); 3 Obstetrics and Gynecology Unit, Villa Sofia Cervello Hospital, I.V.F. Public Center, University of Palermo, Via Trabucco, 180, 90146 Palermo, Italy; gullogiuseppe@libero.it (G.G.); valentina.billone@gmail.com (V.B.); 4 Clinic of Obstetrics and Gynecology Unit, Department of Medical Area (DMED), Santa Maria Della Misericordia Hospital, Azienda Sanitaria Friuli Centrale, 33100 Udine, Italy; restaino.stefano@gmail.com (S.R.); giuseppevizzielli@yahoo.it (G.V.)

**Keywords:** vulvar squamous cell carcinoma, sentinel lymph node biopsy, inguinofemoral lymphadenectomy, previous vulvar surgery, scar injection, recurrence

## Abstract

**Simple Summary:**

According to the latest international guidelines, the standard treatment of primary T1 VSCC < 4 cm consists of wide local excision and SLNB of the inguinofemoral lymph node basin as an alternative standard-of-care approach to lymphadenectomy in select patients. There are many advantages of the SLN procedure compared to lymphadenectomy including a significant reduction in short-term and long-term morbidity. SLNB seems to be feasible, safe, and accurate in patients with a vulvar scar because of a previous excision of the vulvar cancer because it reflects the nodal status and does not negatively impact oncologic outcome. A repeat sentinel node procedure is also possible in patients with local vulvar recurrence after primary treatment who are not able or willing to undergo to lymphadenectomy.

**Abstract:**

Lymphadenectomy for vulvar carcinoma is characterized by many complications. Studies have demonstrated the diagnostic accuracy of sentinel lymph node biopsy (SLNB) as a valid alternative to lymphadenectomy in the early stages of vulvar squamous cell carcinoma (VSCC). **Objective**: To evaluate the feasibility, safety, and accuracy, as well as the oncological outcomes of SLNB following scar injection; in addition, to assess the role of a repeat sentinel node procedure in patients with local vulvar recurrence after primary treatment. **Materials and Methods**: A systematic computerized search of the literature was performed in the main electronic databases (MEDLINE, EMBASE, Web of Science, Pub Med, and Cochrane Library) from 2010 to August 2024. Only scientific publications in English were included. Risk of bias assessment was performed. **Results**: Five articles were included in the study: four retrospective and one prospective observational studies. All patients’ characteristics, including type of surgery, postoperative morbidities, adjuvant therapy, and recurrence, as well as SLN detection and oncological outcomes, have been reported. Four studies compared the scar-injection group (cases) with the tumor-injection group (controls); only one study described the SLNB after vulvar recurrence (second procedure), comparing it with SLNB during primary vulvar surgery (first procedure). **Conclusions**: SLNB is a feasible and safe option in patients who have had previous excision of the vulvar tumor and in patients with a recurrence of VSCC who are not able or willing to undergo lymphadenectomy. Moreover, it accurately reflects the nodal status in these patients.

## 1. Introduction

Vulvar cancer is a rare disease mainly concerning postmenopausal women with a mean age at diagnosis of 68 years that represents approximately 4% of all gynecological neoplasms in the United States of America (USA), with a continuously increasing incidence. The incidence rate is estimated to be 2.5 new cases per 100,000 women per year, similar in Western Europe with 2.4 cases per 100,000 women, and a 20% increase from 1973 to 2000 has been observed in the USA [1,2,3].

Recently, changes have been made to the guidelines for the treatment of vulvar cancer. Surgical excision remains the mainstay of treatment for early-stage tumors, and nodal evaluation is recommended when tumor depth of invasion is >1 mm due to a risk of nodal metastases of at least 7–8% [4,5]. Currently, the standard treatment for unifocal vulvar squamous cell carcinoma (VSCC) measuring 4 cm in diameter without suspected inguinofemoral lymph node invasion on imaging consists of a wide local excision sentinel node biopsy [6,7]. It has been well established that the presence or absence of inguinofemoral lymph node metastasis is one of the most important prognostic factors in vulvar cancer. A subgroup of vulvar squamous cell carcinoma with good prognosis and low risk of metastasis regardless of the type of surgical treatment, pathological features, and the status of surgical margins is the superficially invasive vulvar squamous cell carcinoma (SISCCA). It is defined as a single lesion measuring ≤2 cm with a depth of invasion ≤1.0 mm, where local surgical excision without inguinal lymphadenectomy is a safe and effective treatment but a careful follow-up is necessary, as demonstrated by the retrospective study conducted by Preti et al. [8].

The technique of sentinel node biopsy was first described in the management of cutaneous malignant melanoma, and later by Decesare et al. in patients with invasive squamous cell carcinoma (SCC) of the vulva with clinically negative inguinal nodes [9]. Following this, numerous feasibility studies have established that the technique is accurate, with a negative predictive value close to 100% [4,5,6]. The 2008 GROningen INternational Study on Sentinel nodes in Vulvar cancer (GROINSS-V-I) demonstrated the multiple advantages of the SLN procedure compared to lymphadenectomy, including faster wound healing and the reduced rate of formation of lymphocysts, lymphedema, and recurrent erysipelas, and, therefore, a significant reduction in short-term and long-term morbidity with inguinofemoral SLN biopsy (SLNB) as compared with complete lymphadenectomy [10,11,12,13,14]. Local recurrences, however, are reported in 20–30%, but often is not a question of oncological relapses but of intraepithelial neoplasms (VINs) or lichens [4,5,6]. Inguinofemoral SLNB has been shown to be accurate and safe with a false negative rate of 5%, a negative predictive value of 98%, and a groin recurrence rate of 3% for patients with a negative sentinel lymph node biopsy, but data on the accuracy and safety of repeated SLN procedures are lacking in the literature. There are no current consensus statements or local guidelines on the performance of inguinofemoral SLNB in patients with previous vulvar excision, so the standard treatment in patients with vulvar SCC recurrence is lymphadenectomy [11,12,13,14,15]. The vulvar tumor excision and SLN biopsy are conventionally performed at the same time. Occasionally, in cases where the initial lesion is not suspicious for cancer or when nodal assessment is not recommended, patients firstly have a vulvar tumor resection and then undergo the completion of surgical lymph node staging [16,17,18]. Prior excision may disrupt lymphatic vessels altering the ability to accurately identify SLNs, but studies in other malignancies, such as melanoma, demonstrate reliable identification of the SLN after prior wide local excision [19,20,21]. Studies involving SLN procedures in previously excised vulvar cancer cases have focused on the feasibility of identifying the SLN after injection of the remaining scar.

The objectives of this systematic review are to investigate the feasibility, safety, and accuracy, as well as the oncological outcomes, of SLNB following scar injection; in addition, to assess the role of a repeat sentinel node procedure in patients with local vulvar recurrence after primary treatment who are not able or willing to undergo to lymphadenectomy.

## 2. Materials and Methods

### 2.1. Data Sources

Seven studies were included in a previous version of the review. New studies were identified using electronic databases (MEDLINE, EMBASE, Web of Science, Pub Med, and Cochrane Library) with the use of a mesh combination of the following keywords: “vulvar squamous cell carcinoma”, “sentinel lymph node biopsy”, “inguinofemoral lymphadenectomy”, “previous vulvar surgery”, “scar injection”, and “recurrence”, from 2010 to August 2024. Two authors (L.D.C. and D.B.) independently screened titles and abstracts of studies obtained in the search. All types of studies were selected, and each potentially relevant study was obtained in full text and assessed for inclusion independently by the authors. Disagreements were resolved by consensus with a third reviewer (F.C.). There were five final studies included in the review. The screening of the initial records identified from database is represented in Figure S1.

This article complies with PRISMA guidelines [22]. Table S1 shows the PRISMA 2020 checklist. All references of the retrieved studies were also reviewed to avoid missing relevant publications. Only scientific publications in English were included. All reports related to experimental studies conducted on in vitro or animal models were excluded from the analysis. Proceedings of scientific meetings and abstracts were not considered.

### 2.2. Study Selection

All articles describing SLNB following scar injection were considered for review. Only original papers that reported specific experience data on the topic were included., for a total of 5 studies (Figure S1). Relevant aspects of every article were recorded and commented on, with particular regard to the modality of vulvar cancer treatment. The feasibility, safety, and accuracy, as well as the oncological outcomes, of SLNB following scar injection were evaluated; in addition, the role of a repeat sentinel node procedure in patients with local vulvar recurrence after primary treatment was taken into account. The included patients were divided into cases (SLNB following scar injection) and controls (SLNB during primary surgery).

### 2.3. Risk of Bias

**Figure 1:**
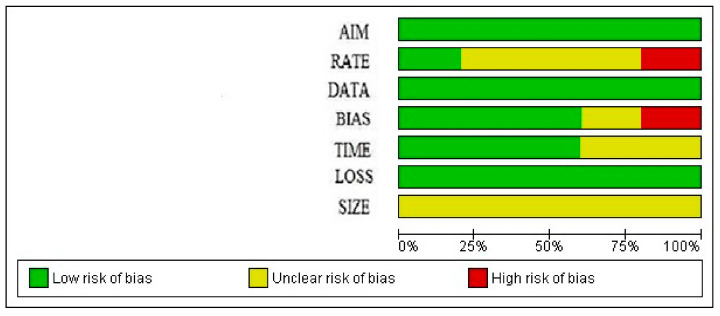
Risk of bias shown graphically.

**Figure 2:**
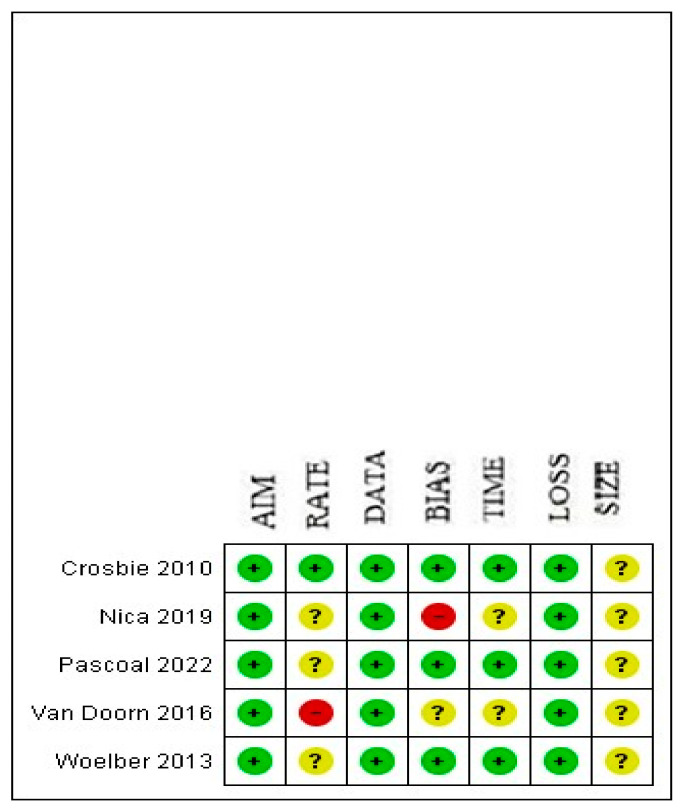
Risk of bias summary [11,12,13,14,15]. (+) low; (-) high; (?) uncertain.

Two authors (L.D.C., D.B.) independently assessed the risk of bias of the included studies via the Methodological Index for Non-Randomized Studies (MINORS), excluding single case reports and video articles. Seven domains related to the risk of bias were assessed in each study: (1) aim (i.e., clearly stated aim), (2) rate (i.e., inclusion of consecutive patients and response rate), (3) data (i.e., prospective collection of data), (4) bias (i.e., unbiased assessment of study endpoints), (5) time (i.e., follow-up time-appropriate), (6) loss (i.e., loss to follow-up), and (7) size (i.e., calculation of the study size). Review authors’ judgments were categorized as “low risk”, “high risk”, or “unclear risk of bias”. Discrepancies were solved by discussion with a third author (F.C.). Low risk was reported for “aim”, “data”, and “loss”, while unclear risk was reported for “size” (Figure 1 and Figure 2).

## 3. Results

### 3.1. Patients’ Selection and Characteristics

From the bibliographic search, a total of 30 articles were retrieved. Twenty-three articles remained after removing duplicates. Seventeen records were assessed for eligibility, then four were excluded because they exclusively dealt with IFL and not SLN biopsy, and then eight were excluded because they exclusively concerned SLN performed during primary vulvar surgery, as explained in Figure S1, using the PRIMA 2020 flow chart. Finally, five studies were included in this systematic review: four retrospective studies [11,12,14,15] and one prospective (observational) study [13], including a total of 295 patients. Figure 2 illustrates the selection of studies for inclusion in the systematic review.

The youngest patient was 20 years old, as reported by Woelber et al. [14], while the oldest was 94 years old, reported by Nica and Crosbie et al. [12,13], with a median age of 63. As analyzed by Nica et al., patients who had SLN detected by scar injection were more likely to be younger (*p* = 0.0001), to have a smaller tumor to start (*p* = 0.002), and to have less depth of invasion (*p* = 0.02) [12]. Also, Pascoal et al. specified that cases (scar-injection patients) had smaller and less invasive tumors compared to the controls (tumor-injection group) [15].

Except for Woelber et al. [14], in all studies, a peri-scar/lesional injection of 99 m Technetium-labeled nanocolloid (preoperative lymphoscintigraphy and intraoperative use of a gamma probe) plus intraoperative blue dye was performed to identify inguinofemoral SLN [11,12,13,15].

After surgery, all patients were followed for a median period of about 35 months (median range of follow-up: 0–118 months) to assess the safety and long-term outcomes of SLN biopsy after previous vulvar surgery. All details regarding patients are summarized in Table 1.

As abovementioned, for all tables, we define “case” as the scar-injection group, while “control” patients are those who underwent concomitant vulvar surgery and inguinofemoral staging.

### 3.2. Tumor Characteristics

Regarding tumor characteristics, Nica, Woelber [34–94], and Pascoal et al. described the FIGO tumor stage with grade 2 as the most represented stadium both for cases and controls [12,14,15], with the exception of cases reported by Pascoal et al., where the most represented was the G1 stage [15]. Lymphovascular space invasion (LVSI) was analyzed by Nica and Pascoal et al. [12,15], and it was negative in the majority of all cases and controls. With regard to the neoplasm side, midline or lateral, the data are very heterogeneous without any specific predominance in all studies, while the tumor size (diameter and depth of lesion in mm) was greater in the control groups than the case ones, with the widest and the deepest reported by Woelber et al. (78 mm of diameter and 27 mm deep) [14]. All details are described in Table 2.

### 3.3. Surgery, Adjuvant Therapy, and Recurrence

In all reviewed studies, all cases and controls underwent wide local excision as vulvar surgery, except in Woelber et al., where 12 cases and 66 controls underwent radical vulvectomy (instead, 20 cases and 8 controls underwent radical local excision) [14]. All data about surgery, adjuvant therapy, and recurrence are represented in Table 3; here, we also report only patients with positive SLNB, while all data regarding this procedure are in Section 3.4.1.

### 3.4. SLN Detection, Postoperative Morbidity, and Oncological Outcomes

#### 3.4.1. SLN Detection

Nica et al. evaluated the accuracy of the SLN procedure after excision biopsy enrolling 24 cases, compared to 106 patients who had SLNB during primary vulvar surgery. SLN was successfully identified in all patients of both groups [12]. The SLNB was negative in 22 (92%) cases and 69 (65%) controls, and positive in 2 (8%) cases and 37 (35%) controls. The cases with positive SLNB underwent systematic inguinofemoral lymphadenectomy (IFL) for a 5 mm metastatic deposit in the SLN. This patient received no adjuvant treatment. The other case with a 2 mm metastatic deposit in the SLN received adjuvant groin radiotherapy without complete dissection [12].

Crosbie et al. analyzed 32 patients with VSCC, 15 cases referred to SLN biopsy after a complete excisional biopsy of the lesion and 17 controls who had a diagnostic tumor biopsy. After preoperative lymphoscintigraphy, SLNs were successfully identified in 97% of patients. Cases showed fewer SLNs on average compared to controls (1.8 vs. 2.6, *p* = 0.03). The procedure accurately detected metastases, which were found in 23% of patients (10 groins), with three patients having bilateral metastases, and one false-negative on one groin [13].

Also, Woelber et al. successfully identified SLNs in all 32 cases and 74 controls, and only 9.4% of the cases showed SLN positivity. Metastasis to the SLNs was observed in 40.5% of controls. All positive cases underwent a complete IFL [14].

SLN detection rates were similar between case and control groups (94% vs. 93%) in Pascoal et al.: only 2 two patients (7.7%) showed SLN positivity, against 14 patients (19.7%) of the controls [15].

Another important point evaluated in these studies was the oncological accuracy and safety of a repeat sentinel node procedure in patients with local vulvar recurrence after primary treatment. Van Doorn et al. reported 27 patients who underwent a repeat SLN procedure, unable or unwilling to undergo lymphadenectomy. The median interval between the first and recurrent disease was 37 months, with a range of 10–146 months. The reasons to omit the (standard) IFL and to perform the alternative treatment with a repeat SLN procedure were frailty and severe co-morbidity in four patients, cognitive impairment in four patients, refusal of IFL in nine patients, and small lesions (<20 mm) at the contralateral site in four patients, while in six patients the reasons could not be retrieved. In the 27 patients with a total number of 44 groins, an SLN procedure was planned. Fifteen of them had a central recurrence and twelve had recurrences away from the midline. SLN detection procedure was successful in 9/15 patients with a central recurrence and in 11/12 patients with recurrences away from the midline (4 bilaterally and 7 ipsilaterally). SLNs were removed from 37/44 groins, so the SLN procedure was feasible in 77% of patients and in 84% of the groins, respectively [11].

#### 3.4.2. Postoperative Morbidity and Oncological Outcomes

Nica et al. followed patients for a median period of 31 months. Overall, 24 patients (18%) developed a recurrence with a median time to recurrence of 9 months. Recurrence occurred in 22 cases of the primary vulvar surgery group. The local recurrence rate in the vulva and the groin was 13% and 5%, respectively. Three cases (3%) presented with a distant or pelvic recurrence and one case (1%) with a synchronous recurrence in the vulva and groin. Other mentioned complications included wound breakdown (11.7%), cellulitis (4.5%), and lymphedema (1.9%). In the scar-injection group, we observed one recurrence (4%) in the groin (Table 3). A lower recurrence rate (4% vs. 22% for the scar-injection group and tumor-injection group, respectively) and a better progression-free survival (PFS) (96% at 1 year) compared to the primary surgery group (86%) were observed. It is mandatory to specify that the patients in the scar-injection group were younger, with smaller and less invasive tumors compared to the control group [12].

No groin recurrences or distant metastases were detected in patients with negative sentinel nodes during the 62 months of median follow-up in Crosbie et al. [13]. Three patients required postoperative radiotherapy to the groins. Four patients (two for cases and two for controls) suffered from vulvar recurrent disease, and other complications included wound infection (3), wound dehiscence (25%), lymphocyst (22%), and chronic lymphedema (16%); two patients of the controls group died. This study did not report results in term of disease, PFS, and recurrence-free survival (RFS) rate because they were not evaluated as primary or secondary objectives.

Woelber et al. followed patients for a median follow-up of 33 months. Recurrence occurred in 13.2% of all patients, with a higher rate in the controls (14.9%) compared to the cases (9.4%). Twenty-five patients underwent radiotherapy, and one chemoradiation. PFS after 24 months was 84% in node-negative patients with SLN biopsy, 92% in node-negative patients who had undergone inguinofemoral lymphadenectomy, and 70% in node-positive patients [14].

During a median follow-up of 34.7 months, recurrence occurred in 21 (29.6%) controls and 4 (15.4%) cases analyzed by Pascoal [15]. In the tumor-injection group, of the four cases who experienced a recurrence, two had local (vulvar) recurrence alone and neither of these patients had undergone scar re-excision, one had recurrence at a distant site, while one of them had combined local, groin, and distant recurrence. Of the 21 controls who developed recurrences, 18 patients had local vulvar recurrences, 5 groin recurrences, and 5 recurrences at a distant site. The cases had fewer recurrences, mainly localized to the vulva. Seven cases and thirty controls underwent radiation after surgery, while six controls underwent chemotherapy. Over the follow-up period, 4 cases and 13 controls died. Groin recurrence rates in patients with negative IF SNLB were not significantly different for the two groups. Two-year recurrence-free survival was 87.5% and 81% for cases and controls, respectively [14].

Finally, sentinel nodes, removed from 37 out of 44 groins in patients with local vulvar recurrence after primary treatment analyzed by Van Doorn et al., resulted positive in four patients (14.8%). Two patients received radiotherapy and two underwent lymphadenectomy. Complications included saphenous vein injuries in three cases and one postoperative vulvar bleeding. Up until that moment, no groin recurrences were observed in a median follow-up of 27 months (range 2–96 months) [11].

Oncological outcomes are summarized in Table 4.

In those studies where they have been reported, although heterogeneous, both scar-injection and tumor-injection groups showed similar overall survival (OS) and PFS rates. However, scar-injection patients showed slightly better PFS outcomes. Tumor size and depth of invasion were consistently the strongest predictors of worse outcomes, with larger and deeper tumors related to higher recurrence and mortality rates. The two-year RFS was a little higher in the scar-injection group. While OS and RFS were comparable in the two groups, the one-year PFS was significantly higher in the scar-injection group compared to the tumor-injection group, and it remained higher in the scar-injection group at five years. Despite the scar-injection group exhibiting better survival outcomes, the difference between the two groups did not reach statistical significance [11,12,13,14,15].

## 4. Discussion

The standard treatment of primary T1 VSCC < 4 cm consists of wide local excision and SLNB of the inguinofemoral lymph node basin as an alternative standard-of-care approach to lymphadenectomy in select patients, according to the recent levels of evidence published by the NCCN Guidelines Version 1.2024 Vulvar Neoplasms [7]. Lymph node metastasis is the most important prognostic factor in patients with VSCC [16]. All the included studies evaluated the role of SLN biopsy in patients with and without previous excision of the vulvar tumor. The findings from included studies provide important insights into the feasibility, accuracy, and safety of the SLN procedure in patients with prior vulvar excisions and consequent scar injection for VSCC, even in case of vulvar recurrence.

Nica et al. demonstrated that scar injection can reliably identify the inguinofemoral SLN, confirming that lymphatic mapping is not significantly compromised by prior surgeries [12]. Similarly, Crosbie et al. and Woelber et al. echoed this conclusion, reporting SLN detection rates of 96–97% in patients with previously excised vulvar tumors [13,14]. Pascoal et al. analyzed the vulvar recurrence rate in the scar-injection group, which was significantly lower than that in the tumor-injection group (15.4% vs. 29.6%), with no groin recurrences after a median follow-up of 34.7 months [15]. Also, Nica et al. found a low rate of recurrence, with only one groin recurrence, in the scar-injection group [12]. These findings suggest that scar injection ensures accurate SLN identification. In addition, the feasibility of a repeat SLN procedure in recurrent VSCC was explored by van Doorn et al., with successful identification in 77% of patients and 84% of groins [11].

In patients with recurrent VSCC, IFL is considered the standard treatment for patients who previously did not undergo it. Since many of these patients are elderly and frail, an alternative treatment to IFL may be justified to avoid or reduce long-term morbidity. The procedure is technically more challenging in the recurrent setting, with SLNs found in atypical locations, sometimes outside standard inguinofemoral boundaries [17,18]. Despite these challenges, no groin or distant recurrences were observed in patients with negative SLNs during a median follow-up of 27 months [11].

As abovementioned, the most important issue analyzed in all the reviewed studies was the possible altered lymphatic drainage pathways in patients who had undergone prior surgery, which can make SLN mapping more difficult. The same problem was also observed in melanoma and breast cancer patients by Estourgie et al., Ariyanv et al., and Gannon et al. [19,20,21], where abnormal lymphatic pathways were documented after previous surgeries.

As for breast cancer and melanoma, preoperative lymphoscintigraphy is used to visualize the lymphatic drainage pattern and location of the sentinel node in women with VSCC. Estourgie et al. reported how lymphatic mapping cannot be completed accurately after excision biopsy because it is not clear whether the sentinel node identification rate and the consequent false-negative rate are affected by previous excision biopsy. In that study, lymphoscintigraphy performed at baseline was compared with a lymphoscintigraphy performed 2 weeks after the surgery, repeated by the same investigator. The two sets of images were independently compared by a panel of two experienced observers concerning similarity of the following variables: lymphatic flow pattern, draining lymph node basin, location, and number of sentinel nodes. A discrepancy was reported in the drainage pattern in a total of 17/25 scans, with a rate of reproducibility of the overall lymphoscintigraphic of 32%. However, these data could also be supported by the radiotherapy administered to the patients, which could have sterilized the involved lymph nodes remaining in the armpit [19]. Aryan et al. analyzed the preoperative lymphoscintigraphy of 20 patients with melanoma, with the procedure performed 2–4 weeks after the surgery in 19 patients (1 patient declined to undergo postoperative lymphatic mapping), and found an identical preoperative mapping in 13 patients (68%), additional lymph nodes in 4–5 patients (21–26%), and fewer lymph nodes in 1–2 patients (5–10%) [20]. Also, Gannon et al. evaluated the same issue on patients surgically treated for melanoma, and the results were superimposable [21]. Lymphatic mapping performed after the excision of a primary cutaneous melanoma should be as reliable in identifying the SLN as a preoperative mapping in 90% of the patients, and does not appear to adversely impact the ability to detect lymphatic metastases, although the utility remains to be defined [19,20,21], while there are conflicting opinions for the breast cancer [19].

Van Doorn et al.’ study deserves a separate chapter: they carried out another study about the repeat sentinel node procedure and proposed a protocol for a multicenter study to be performed in five hospitals in the Netherlands [23]. The aim was to investigate the safety and feasibility of the SLN procedure in the treatment of recurrent V-SCC. The primary objective of their study was to determine the percentage of women who develop an inguinal recurrence within 24 months of a technically successful SLN procedure. According to the protocol, all patients with a first recurrence of vulvar cancer, specifically those with a unifocal tumor smaller than 4 cm, without distant or inguinal metastases, were selected to replace the IFL procedure with SLN. The authors established the inclusion criteria for these patients: previous surgical treatment with wide local excision or partial vulvectomy; tumor size equal to or less than 4 cm, not invading the urethra, vagina, or anus; tumor location permitting perilesional injection of tracers at three or four sites; clinically negative inguinofemoral lymph nodes; and preoperative imaging demonstrating no enlarged (>10 mm of the selection axis) or suspicious lymph nodes. Using software (https://biostatistics.mdanderson.org/SoftwareDownload/SingleSoftware/Index/84, accessed on 1 February 2025), the authors calculated that the minimum number of patients needed to achieve adequate statistical power was 150, all with a first recurrence of vulvar cancer, who underwent a negative SLN procedure. This implies that approximately 240–250 women would be enrolled in the study, since the SLN procedure should fail in approximately 25–30% of cases and the SLN should be positive for cancer in 8–10% of cases. If the SLN procedure proves safe and feasible in this group of patients, it could significantly contribute to reducing the short- and long-term side effects of vulvar cancer treatment, while improving quality of life compared to the current standard of care [23]. Although this is an ongoing study, it offers new hope and future prospects for the use of this technique which still presents many questions today.

Our review has some limitations, such as the few studies published in the literature so far, the small sample size, the retrospective design of four studies, and the heterogeneity of the presented data (patients [12,13,14,15] and/or groins [11]), which limited the scope to draw definitive conclusions. Woelber et al. pointed out the “positive selection” of patients in the scar-injection group, where tumors with less aggressive clinical features might have influenced the results [14]. Crosbie et al. highlighted potential challenges in patients with midline tumors or, indeed, previous excisions, where SLN detection might be less reliable. The false-negative rate observed in a patient with a previously excised midline tumor underscores the need for careful consideration in these cases [13]. Also, Nica et al. highlighted that tumors in the scar-injection group were generally smaller and less invasive, potentially affecting recurrence and survival outcomes [12]. Although this is a qualitative review, the strength of this study is linked to the careful analysis of the cases compared to controls analyzed in the five studies.

## 5. Conclusions

This review suggests that the SLNB procedure is a safe and effective option for the management of VSCC, even in patients who have already undergone vulvar excision. When SLN is detected by injecting the remaining scar, this accurately reflects the nodal status and does not negatively impact oncologic outcome. A repeat sentinel node procedure is also possible in patients with local vulvar recurrence after primary treatment who are not able or willing to undergo to lymphadenectomy. In patients with recurrent VSCC, IFL is considered standard treatment for patients who previously did not undergo an IFL because elderly and frail and because SLNs is often found in atypical sides: despite these challenges, in the included studies, no groin or distant recurrences were observed in patients with negative SLNs during the follow-up period. Patients treated with scar injection showed lower rates of local and groin recurrence, suggesting that this technique may be preferable for cases involving smaller or less invasive tumors. We are confident about the results that the new study protocol proposed by Van Doorn et al. [23] can aspire to determine the percentage of women who develop a groin recurrence after a technically successful SLN procedure, investigating its safety and feasibility in the treatment of recurrent V-SCC.

Future studies should focus on directly comparing SLN biopsy with scar injection versus tumor injection, ideally through randomized prospective trials with larger cohorts. Additional research is needed to better understand how prior surgery affects lymphatic mapping, because there are few data in the literature regarding this problem for vulvar cancer. Until more robust data are available, careful patient selection and thorough preoperative assessment are crucial to ensure accurate SLN identification and minimize the risk of false negatives.

## Figures and Tables

**Table 1:** General characteristics of included studies (na: not applicable). * 15 patients received an excisional biopsy (cases group) while 17 received a diagnostic biopsy with the primary vulval lesion still in situ (controls group) but there is not a subclassification for the age of the patients, duration of follow-up, and treatment-related morbidity (case or control that they were). ** 32 patients had vulvar surgery with wide local excision before SLN (cases group) and 74 patients received both procedures during primary surgery (controls group), but there is not a subclassification for the age of the patients, for duration of follow-up, and for treatment-related morbidity if they are case or control. *** 27 is the same number of patients considering the first procedure and the repeated SLNB after recurrence; 39 and 15 are the number of groins of the first and second procedure, respectively, of a total of 27 patients who were not able or willing to undergo inguinofemoral lymphadenectomy as part of their treatment for recurrent vulvar cancer, so they received a repeat SLN procedure. ^ Cases group included patients who underwent SLNB by scar injection, while the controls one included patients who had injection of technetium/blue dye around visible tumor. ° A further 2 patients had a lateralized tumor treated with wide local excision and had negative unilateral SLN biopsy: they were subsequently found to have a new primary tumor lateralized to the contralateral vulva, and were again treated with a wide local excision and had negative unilateral SLN biopsy.

Study	Country	Type of Study	Patients		Age (Years) Median [Range]		Duration of Follow-Up (Months) Median [Range]	
			Cases (*n*)	Controls (*n*)	Cases	Controls	Cases	Controls
Crosbie et al. (2010) [13]	United Kingdom	Prospective (observational) study	15 *	17 *	67 [34–94] *		62 [33–84] *	
Woelber et al. (2012) [14]	Germany	Retrospective (monocentric) study	32 **	74 **	57.5 [20–87] **		33 [3–118] **	
Van Doorn et al. (2016) [11]	The Netherlands	Retrospective cohort study	27 ***	7 ***	65.3 [35–87] ***		27 [2–96]	
Nica et al. (2019) [12]	Canada	Retrospective (monocentric) study	24 ^°	104 ^°	53 [31–78]	68 [38–94]	22 [0.03–96]	31 [0.3–103]
Pascoal et al. (2022) [15]	Canada	Retrospective (observational) cohort study	26	71 ^	61.6 [46.3–76.9]	66.4 [52.7–80.1]	34.7 [0–108]	

**Table 2:** VSCC (vulvar squamous cell carcinoma) characteristics (mid: midline; lat: lateral; nr: not reported; LVSI: lymphovascular space invasion; * cases correspond to patients with scar injection, while controls correspond to patients with tumor injection; ^ in 27 patients, a repeat SLN procedure was performed).

Study	Diameter of Tumor (mm) [Median (Range)]		Depth (mm) [Median (Range)]		Vulvar Tumor Side *n* (%)				Grade (%) [G1–G2–G3]		LVSI (%) [Negative–Positive]	
	Case	Control	Case	Control	Case		Control		Case	Control	Case	Control
					Mid	Lat	Mid	Lat				
Crosbie et al. (2010) [13]	<40	<40	>1	>1	7 (46.6%)	8 (53.3%)	10 (58.8%)	7 (41.1%)	nr	nr	nr	nr
Woelber et al. (2012) [14]	9 (2.5–28)	19 (1–75)	2 (1–11)	4 (0.8–27)	nr	nr	nr	nr	G1 = 9.7% G2 = 74.2% G3 = 16.1%	G1 = 8.1% G2 = 64.9% G3 = 27%	nr	nr
Van Doorn et al. (2016) [11]	13 (2–65) ^	13 (4–31) ^	3 (1.1–10) ^	3.5 (1.0–10) ^	15 (55.5%) ^	12 (44.4%) ^	12 (44.4%) ^	15 (55.5%) ^	nr	nr	nr	nr
Nica et al. (2019) [12]	8 (4.0–12.0)	13 (7.8–22.0)	2.7 (1.5–4)	4.3 (2.2–6.5)	10 (42%)	14 (58%)	67 (63%)	39 (37%)	G1 = 50% G2 = 50% G3 = 0%	GI = 37% G2 = 51% G3 = 12%	92–8%	87–13%
Pascoal et al. (2022) [15]	13.4 (2.5–24.3) *	27.8 (14.8–40.8) *	3.7 (0.6–6.8) *	5.6 (2–9.2) *	17 (65.4%) *	9 (34.6%) *	57 (80.3%) *	14 (19.7%) *	G1 = 50% G2 = 44% G3 = 6%	G1 = 36% G2 = 62% G3 = 2%	92.3–7.7%	80.2–19.7%

**Table 3:** Surgery, postoperative morbidities, adjuvant therapy, and recurrence. ° The cases group included patients who underwent SLNB by scar injection, while the controls one included patients who had injection of technetium/blue dye around visible tumor. * 15 patients received a radical local excision of vulval scar after an excisional biopsy (cases), while 17 received a radical local excision after a diagnostic biopsy (controls). # Compared to others, this study specified patients with positive groins. ᶞ Not specified whether in the case or control group. ^¥^ Not specified whether it is among the cases or the controls. ** Of the 4 patients who experienced a recurrence in the scar-injection group, 2 had local (vulvar) recurrence alone, 1 patient had a distant recurrence, and 1 had a combined local, groin, and distant recurrence; of the 21 patients in the tumor-injection group who developed recurrence, 18 patients (85.7%) had local (vulvar) recurrences, 5 (23.8%) had groin recurrences (3 of whom had negative sentinel lymph node biopsy), and 5 (23.8%) had recurrence at a distant site). ^ SLNB was successful in 9/15 patients with a central recurrence and in 11/12 patients with recurrences away from the midline; out of 27 patients, only 4 of them had positive SLN.

Study	Type of Surgery ° *n* (%)				Patients with Positive SLN Biopsy *n* (%)		Treatment-Related Morbidity *n* Patients (%)	Adjuvant Radiotherapy *n* (%)			Recurrence *n* (%)							
	Case		Control															
	Vulvectomy	Radical Re-Excision	Vulvectomy	Radical Excision	Case	Control		Case		Control	None		Vulva/Local		Groin		Distant	
											Case	Control	Case	Control	Case	Control	Case	Control
Crosbie et al. (2010) [13]	0	15 (46.8%) *	0	17 (53%) *	2 (6.2%) #	5 (15.6%) #	Wound infection 10 (31%) Wound dehiscence 8 (25%) Lymphocyst 7 (22%) Chronic lymphedema 5 (16%) °	3 (9.3%) ^¥^			0		2 (13.3%)	2 (11.7%)	1 (3.1%) ᶞ		0	
Woelber et al. (2012) [14]	12 (37.5%)	20 (62.5%)	66 (89%)	8 (10.8%)	3 (9.4%)	30 (40.5%)	nr	1 (3.1%)		19 (26%)	29 (90.6%)	63 (85.1%)	3 (9.4%)	6 (8.1%)	0	4 (5.4%)	1 (1.4%)	0
Van Doorn et al. (2016) [11]	0	27 (100%)	0	27 (100%)	4 (14.8%) ^	na	Postoperative groin complications 4 (14%) and lesion of the saphenous vein in *3* groins	2 (7.41%) ^¥^			23 (85%)	nr	4 (14.8%)	nr	0	0	0	0
Nica et al. (2019) [12]	0	24 (100%)	0	104 (100%)	2 (8%)	37 (35%)	Wound dehiscence (11.7%) Cellulitis (4.5%) Lymphedema (1.9%)	0	12 (11%)		23 (96%)	83 (78%)	0	14 (13%)	1 (4%)	5 (5%)	0	3 (3%)
Pascoal et al. (2022) [15]	0	26 (100%)	0	71 (100%)	2 (7.7%)	14 (19.7%)	nr	7 (26.9%)	30 (42.2%)		22 (84.6%) **	50 (70%) **	3 (75%) **	18 (85.7%) **	1 (25%) **	5 (23.8%) **	1 (25%) **	5/21 (23.8%) **

**Table 4:** Oncological outcomes. nr: not reported; ° Time in months is expressed as a median, except for Pascoal et al. [15], who used a specific time of 24 months (not intended as median). * Cases correspond to patients with scar injection, while controls correspond to patients with tumor injection. ^§^ Median follow after 2nd SN procedure with no recurrence so far.

Study	Disease-free Survival % Patients (Months) *°*		Recurrence-free Survival % Patients (Months) *°*		Overall Survival % Patients (Months) *°*	
	Cases	Controls	Cases	Controls	Cases	Controls
Crosbie et al. (2010) [13]	nr		nr		nr	
Woelber et al. (2012) [14]	92.5 (105) *	72.5 (80) *	nr		nr	
Van Doorn et al. (2016) [11]	nr		27 months (range 2–96) ^§^		nr	
Nica et al. (2019) [12]	96 (38)	86 (26)	nr		nr	
Pascoal et al. (2022) [15]	nr		87.5 (24)	81 (24)	87 (24)	88 (24)

## Data Availability

Data are contained within the article.

## Supplementary Materials

The following supporting information can be downloaded at: https://www.mdpi.com/article/10.3390/cancers17040673/s1, Figure S1: Flow diagram of systematic review search (PRISMA). IFL: inguinofemoral lymphadenectomy; SLN: sentinel lymph node; Table S1: PRISMA 2020 checklist.
