# Supplementary material for: Is the Sentinel Lymph Node Biopsy Safe and Accurate After Previous Surgery for Vulvar Squamous Cell Carcinoma? A Systematic Review
Source: Cancers (Basel). 2025 Feb 17;17(4):673. doi: 10.3390/cancers17040673 (PMC11853356; doi:10.3390/cancers17040673)
Supplement: Supplementary file 1 [file cancers-17-00673-s001.zip › cancers-3448151-supplementary.pdf]

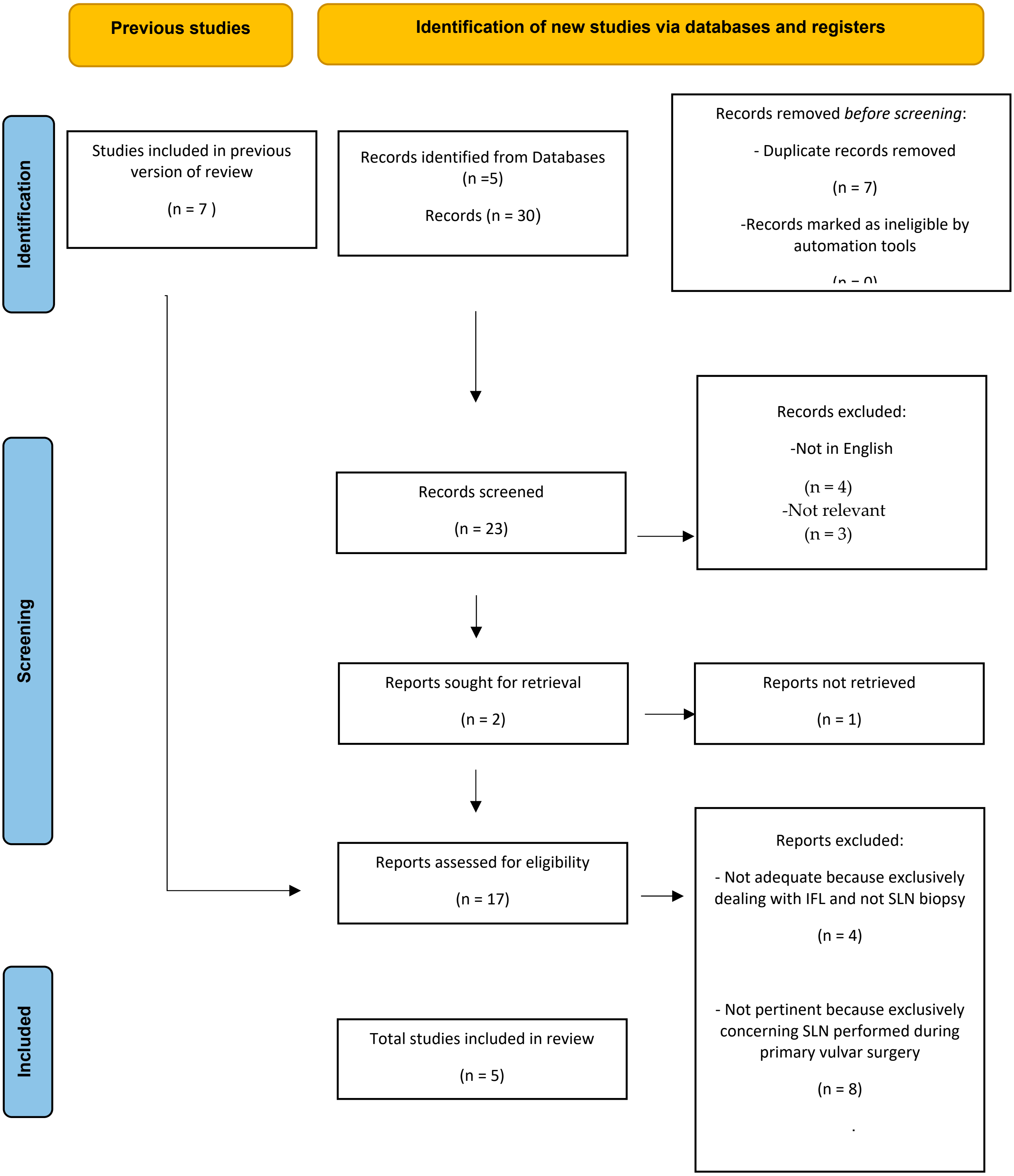

Figure S1. Flow diagram of systematic review search (PRISMA). **IFL:** inguinofemoral lymphadenectomy; **SLN:** sentinel lymph node.

Table S1. PRISMA 2020 checklist.

Section and Topic	Item #	Checklist item	Location where item is reported
**TITLE**			
Title	1	Is the sentinel lymph node biopsy safe and accurate after pre-vious surgery for vulvar squamous cell carcinoma? A systematic review.	Line 103
**ABSTRACT**			
Abstract	2	Lymphadenectomy for vulvar carcinoma is characterized by many complications. Studies have demonstrated the diagnostic accuracy of sentinel lymph node biopsy (SLNB) as a valid alternative to lymphadenectomy in the early stages of vulvar squamous cell car-cinoma (VSCC). Objective: to evaluate the feasibility, safety and accuracy as well as the oncological outcomes of SLNB following scar injection; in addition, to assess the role of a repeat sentinel node procedure in patients with local vulvar recurrence after primary treatment. Materials and Methods: a systematic computerized search of the literature was performed in the main electronic databases (MEDLINE, EMBASE, Web of Science, Pub Med, and Cochrane Library), from 2010 to August 2024. Only scientific publications in English were included. Risk of Bias Assessment was performed. Results: 5 articles were included in the study: 4 retrospective and 1 prospective observational studies. All pa-tients' characteristics, including type of surgery, post-operative morbidities, adjuvant therapy and recurrence as well as SLN detection and oncological outcomes have been reported. Four studies compared the scar-injection group (cases) with the tumor-injection group (controls); only one study (Van Doorn et al.) described the SLNB after vulvar re-currence (second procedure) comparing it with SLNB during primary vulvar surgery (first procedure). Conclusions: SLN biopsy is feasible and safe option in patients who have had previous excision of the vulvar tumor and in patients with a recurrence of VSCC who are not able or willing to undergo lymphadenectomy. Moreover, it accurately reflects the nodal status in these patients.	Line 29-48
**INTRODUCTION**			
Rationale	3	The vulvar tumor excision and SLN biopsy are conventionally performed at the same time. Occasionally, in cases where the initial lesion is not suspicious for cancer or when nodal assessment is not recommended, firstly patients have a vulvar tumor resection and then the completion of surgical lymph node staging. Prior excision may disrupt lymphatic vessels altering the ability to accurately identify SLNs, but studies in other malignancies such as melanoma demonstrate reliable identification of the SLN after prior wide local excision. Studies involving SLN procedures in previously excised vulvar cancer cases have focused on the feasibility of identifying the SLN after injection of the remaining scar.	Line 94-102
Objectives	4	The objectives of this systematic review are to investigate the feasibility, safety and accuracy as well as the oncological outcomes of SLNB following scar injection; in addition, to assess the role of a repeat sentinel node procedure in patients with local vulvar re-currence after primary treatment who are not able or willing to undergo to lymphade-nectomy	Line 103-107
**METHODS**			
Eligibility criteria	5	All articles describing SLNB following scar injection were considered for review. Only original papers that reported specific experience data on the topic and only scientific publications in English were included	Line 122, 125-127
Information sources	6	Studies were identificated using the following electronic databases (MEDLINE, EMBASE, Web of Science, Pub Med, and Cochrane Library	Line 110-111
Search strategy	7	We use a mesh combination of the following keywords "vulvar squamous cell carcinoma", "sentinel lymph node biopsy", "inguinofemoral lymphadenectomy", "previous vulvar surgery", "scar injection" and "recurrence"	Line 112-114
Selection process	8	All references of the retrieved studies were reviewed to avoid missing relevant publications. All reports related to experimental studies conducted on in vitro	Line 122-124

Section and Topic	Item #	Checklist item	Location where item is reported
		or animal models were excluded from the analysis. Proceedings of scientific meetings and abstracts were not considered.	
Data collection process	9	Two authors (LDC and DB) independently screened titles and abstracts of studies obtained in the search. All types of studies were selected and each potentially relevant study was obtained in full text and assessed for inclusion independently by the authors. Disa-greements were resolved by consensus with a third reviewer (FC)	Line 115-118
Data items	10	All results compatible with each outcome domain in each study were searched for.	Line 110
Study risk of bias assessment	11	Two authors (LDC, DB) independently assessed the risk of bias of the included studies via the Methodological Index for Non-Randomized Studies (MINORS), excluding single case reports and video articles.	Line 189-191
Effect measures	12	Seven domains related to the risk of bias were assessed in each study: (1) aim (ie clearly stated aim), (2) rate (ie inclusion of consecutive patients and response rate), (3) data (ie prospective collection of data), (4) bias (ie unbiased assessment of study endpoints), (5) time (ie follow-up time-appropriate), (6) loss (ie loss to follow up), (7) size (ie calculation of the study size).	Line 191-198
Synthesis methods	13	Relevant aspects of every article were recorded and commented, with particular regard to the modality of vulvar cancer treatment. The feasibility, safety and accuracy as well as the oncological outcomes of SLNB following scar injection have been evaluated; in addition, the role of a repeat sentinel node procedure in patients with local vulvar recurrence after primary treatment has been taken into account. The included patients have been divided into cases (SLNB following scar injection) and controls (SLNB during primary surgery). No meta-analysis has been performed.	Line 129-135
Reporting bias assessment	14	Review authors' judgments were categorized as "low risk", "high risk" or "unclear risk of bias". Discrepancies were solved by discussion with a third author (FC). Low risk was reported for "aim", "data" and "loss" while unclear risk was reported for "size".	Line 195-198
Certainty assessment	15	Only original papers that reported specific experience data on the topic were included.	Line 128-129
**RESULTS**			
Study selection	16	From the bibliographic search, a total of 30 articles were retrieved. Twenty-three articles remained after removing duplicates. 17 records were assessed for eligibility, and then 4 were excluded because exclusively dealing with IFL and not SLN biopsy and 8 were excluded because exclusively concerning SLN performed during primary vulvar surgery.	Line 206-212
Study characteristics	17	5 studies were included in this systematic review: 4 retrospective studies and 1 prospective (observational) study	Line 213-214
Risk of bias in studies	18	Risk of bias of included studies is represented in Figure 2.	Line 202
Results of individual studies	19	Results of individual studies are represented in Table 4.	Line 404
Results of syntheses	20	No meta-analysis has been performed.	-
Reporting biases	21	Risk of bias of included studies is represented in Figure 2.	Line 202
Certainty of evidence	22	Only original papers that reported specific experience data on the topic were included.	Line 128-129
**DISCUSSION**			
Discussion	23	The standard treatment of primary T1 VSCC <4 cm consists of wide local excision and SLNB of the inguinofemoral lymph node basin as an alternative standard-of-care ap-proach to lymphadenectomy in select patients, according to the recent levels of evidence published by the NCCN Guidelines Version 1.2024	Line 421-493

Section and Topic	Item #	Checklist item	Location where item is reported
		Vulvar Neoplasms. Lymph node metastasis is the most important prognostic factor in patients with VSCC. All the included studies evaluated the role of SLN biopsy in patients with and without previous excision of the vulvar tumor. The findings from included studies provide important in-sights into the feasibility, accuracy, and safety of the SLN procedure in patients with prior vulvar excisions and consequent scar injection for VSCC, even in case of vulvar recurrence. Our review has some limitations, such as the small sample sizes, the retrospective design of four studies and the heterogeneity of presented data patients and/or groins which limited the scope to draw definitive conclusions. Woelber et al. pointed out the "positive selection" of patients in the scar-injection group, where tumors with less aggressive clinical features might have influenced the results. Crosbie et al. highlighted potential challenges in patients with midline tumors or indeed previous excisions, where SLN detection might be less reliable. The false-negative rate observed in a patient with a previously excised midline tumor underscores the need for careful con-sideration in these cases. Also, Nica et al. highlighted that tumors in the scar-injection group were generally smaller and less invasive, potentially affecting re-currence and survival outcomes. Although this is a qualitative review, the strength of this study is linked to the careful analysis of the cases compared to controls analyzed in the 5 studies.	
**OTHER INFORMATION**			
Registration and protocol	24	Conceptualization, L.D.C. and D.B.; methods, D.B. and F.C..; writing—original draft preparation, D.B. and F.C.; review and editing L.D.C. and D.B; validation L.D.C., G.G., V.B., S.R., G.V., P.G. and G.B; supervision G.B. All authors have read and agreed to the published version of the manu-script. Institutional Review Board Statement not applicable. Informed Consent Statement not applicable.	Line 516-519
Support	25	This research received no external funding.	Line 520
Competing interests	26	All authors declare that there are no conflicts of interest involved with the presented data. This study was funded by internal departmental sources	Line 524-525
Availability of data, code and other materials	27	Data Availability Statement not applicable.	Line 523
